# Animal and human innovation: novel problems and novel solutions

**DOI:** 10.1098/rstb.2015.0182

**Published:** 2016-03-19

**Authors:** Simon M. Reader, Julie Morand-Ferron, Emma Flynn

**Affiliations:** 1 Department of Biology, McGill University, 1205 avenue Docteur Penfield, Montreal, Quebec, Canada H3A 1B1; 2 Department of Biology, University of Ottawa, Ottawa, Ontario, Canada K1N 6N5; 3 School of Education, Durham University, Durham DH1 1TE, UK

**Keywords:** behavioural innovation, social learning, culture, intelligence, evolution, invention

## Abstract

This theme issue explores how and why behavioural innovation occurs, and the consequences of innovation for individuals, groups and populations. A vast literature on human innovation exists, from the development of problem-solving in children, to the evolution of technology, to the cultural systems supporting innovation. A more recent development is a growing literature on animal innovation, which has demonstrated links between innovation and personality traits, cognitive traits, neural measures, changing conditions, and the current state of the social and physical environment. Here, we introduce these fields, define key terms and discuss the potential for fruitful exchange between the diverse fields researching innovation. Comparisons of innovation between human and non-human animals provide opportunities, but also pitfalls. We also summarize some key findings specifying the circumstances in which innovation occurs, discussing factors such as the intrinsic nature of innovative individuals and the environmental and socio-ecological conditions that promote innovation, such as necessity, opportunity and free resources. We also highlight key controversies, including the relationship between innovation and intelligence, and the notion of innovativeness as an individual-level trait. Finally, we discuss current research methods and suggest some novel approaches that could fruitfully be deployed.

## 1. Introduction

Innovation is a key characteristic of human life and of the success of our species. Culturally transmitted innovations allow humans to survive and prosper in the toughest environments, we have extended lifespan through the creation of medicines, vaccines and improved sanitation, and our very societies and social networks are themselves constructed by social and technological innovations [1,2]. Such innovation has also had costs and raised new challenges, from technological competition in business, to exposure to novel pathogens, and to literal arms races. The importance of innovation to human life is reflected in the numerous institutions and programmes devoted to promoting innovation, and it is not surprising that human innovation has a rich history of research, with contributions from fields as diverse as anthropology, archaeology, economics, psychology, philosophy and sociology [2–8].

Innovation is also widespread in non-human animals (henceforth, ‘animals’), and evidence is mounting for its adaptive importance [9,10]. Innovation is thought to play important roles in animal ecology and evolution, for instance facilitating range expansion and subspecies diversification, and is a vital first step of social learning and cultural diversification. Animal innovations appear in both the social domain (e.g. new song variants, novel mating and dominance displays, some instances of tactical deception) and the non-social domain (e.g. novel tool use, diets or foraging techniques) [9,10]. Like human innovation, research on innovation in non-human animals has a rich history (for review, see [11]). Key in the development of interest in animal innovation were early papers identifying the appearance and spread of novel behaviour patterns in wild populations, particularly in birds, primates and cetaceans (e.g. [12–16]), as well as suggestions that innovation could shape evolutionary processes (e.g. [17,18]). Large-scale surveys taking advantage of the rich ornithological literature established that innovations were taxonomically widespread, rather than performed by a few select individuals or species, setting the stage for investigations of evolutionary patterns and how innovative propensities evolved [19,20]. Also relevant was work on tactical deception in primates, which identified many novel behaviour patterns [21,22]. However, research on animal innovation has been more limited in scope than that on human innovation, and it is only relatively recently that animal innovation has been identified as a field of research [9], although many related fields of work touch on behavioural innovation, such as research on phenotypic plasticity and social learning [23,24]. Interest in animal innovation has grown rapidly, as evidenced by edited volumes, reviews, models and commentaries [9,10,25–28], as well as numerous empirical articles (see e.g. [29–31] for review).

However, at first sight, there is a vast gulf between the foraging innovations of birds and primates and the incredible complexity of human creativity, as exemplified by our computers, satellites and particle accelerators. Yet, the capacity for human innovation must itself have evolved. Why and how were human innovative capabilities favoured by selection? Did these capacities develop and evolve independently, or as part of a suite of traits, or even as an emergent property of other traits? This theme issue explores both human and animal innovation to examine whether useful links can be made between these domains of research. We bring together authorities on human innovation, childhood creativity and animal innovation, to promote an up-to-date interdisciplinary dialogue. By inviting experts in philosophy of science, anthropology, developmental psychology, behavioural biology and evolutionary biology, we provide a forum for the exchange of methods, theory and paradigms. Yet with this cross-disciplinary dialogue comes misalignment and disagreement. In this brief introduction, we highlight points of consensus and disparity and make links to the wider field.

Cross-disciplinary dialogue is important, because a deeper understanding of innovation, in terms of its antecedents, development, transmission and consequences, has clear practical and social implications. Economic growth and scientific progress both demand constant innovation. Rapidly changing environments (derived from, for instance, climatic change, economic crises or depleting resources) mean that humans, and often other species too, must be adaptable. However, innovation carries risks, and findings in humans and animals suggest that innovation can have significant costs [6,32–34]. For example, a bias towards innovation means that companies frequently over-invest in innovation, risking bankruptcy, when circumstances dictate that maintaining the status quo or imitating other firms would be a more effective strategy [35]. Establishing what underpins successful innovation, and isolating which processes and conditions facilitate and which impede it, has wide-reaching implications for issues such as the development of new technologies, tracking change effectively and the avoidance of maladaptive behaviour by endangered species. A current concern, for example, is that modern childhood may curtail the development of creative thinking through over-instruction, pressure to follow social norms and a lack of free time to explore, and thus potentially undermine innovation [36]. Isolating the contexts in which innovation occurs potentially allows society to promote innovation by facilitating the processes that underpin an individual's, or group's, ability to design and evaluate alternatives (e.g. this issue: [37]). These issues are equally important for other animals. There is now comparative evidence that innovative species are more likely to survive in new locations compared to less innovative species, and within-species evidence linking innovation to fitness measures [38–41]. This theme issue provides a valuable step towards the longer term possibility of constructing environments and designing interventions to facilitate innovation where appropriate, and, indeed, establishing when and what kind of innovation is appropriate.

## 2. Categorizing and defining innovation

A recurring controversy within and between fields is how to adequately define innovation (for extensive discussion see [11,25,42,43] and accompanying commentaries). Indeed, some authors have raised the concern that the attempt to over-define innovation can stifle, rather than increase progress [44]. We certainly agree that definitional arguments can provide more heat than light, particularly in the absence of knowledge about the processes that underlie innovation or the functional consequences of different types of innovation. Nonetheless, some agreement over definitions is important to effective dialogue, both within and between subfields, and to allow those outside the field to grasp the phenomenon under investigation. Agreement over definitions is particularly problematic given the comparative nature of our enterprise and the range of behaviour under investigation: what definition can sensibly capture a novel scientific theory, a 5-year-old child struggling to solve a new task easily mastered by 8-year-olds, and an animal shifting to a new food, host or foraging technique?

Building on previous treatments (e.g. [15,17,45]), Reader & Laland [11] proposed two definitions, distinguishing between innovation as a product and a process. An innovation (*sensu* product) is ‘a new or modified learned behaviour not previously found in the population’, while innovation (*sensu* process) is ‘a process that results in new or modified learned behaviour and that introduces novel behavioural variants into a population's repertoire’ (p. 14). These definitions were explicitly operational and designed to identify innovations in natural, free-living populations, rather than novel behaviour evoked by experimental testing. Innovations were defined as ‘learned’ to eliminate accidental occurrences and to focus on behavioural variants likely to be functionally important to the individual (since unimportant behaviours would presumably not be retained in the innovator's repertoire). While two individuals clearly can independently invent the same behaviour, only the first instance would count as innovation under the current definition. This stance thereby distinguishes innovation from other learned behaviour. The ‘not previously found in the population’ criterion is an attempt to operationalize the difficult question of what is sufficiently novel to ‘count’ as innovation [46,47], although ‘not previously found’ is still open to interpretation and possible bias. Comparative studies of innovation that rely on spontaneous innovations have thus implemented methods to address such possible biases (see below). The population-level focus is crucial for such studies since counting repeated cases of an innovation would lead to inflated innovation rates in taxa where the social learning of innovations is frequent. However, it should be noted that different definitions may be appropriate when using an experimental approach [42,43] where individuals or groups are presented with novel problem-solving tasks (see §3).

When considering innovation, authors in this theme issue [18,34,48–50] and beyond [25,43,51–55] have highlighted the possibility for further delineation. Sub-categorizing innovation can facilitate a deeper understanding of the behaviour under investigation. For example, some innovation may occur through chance, such as learning as a result of accidental acts, copying error or natural occurrences. These have been labelled passive [56], type II [57] or low level [43], while other innovations seemingly occur through causal inference and deliberate action, sometimes called complex [58], type I [57] or active [56]. Equally, researchers such as Mesoudi *et al.* [55] have looked at the many forms of innovation possible through individual or group endeavour, including novel invention (produced by trial and error, insight or exploration), refinement (modification or improvement of existing variants), recombination (combining existing elements to form a new variant) and exaptation (reuse beyond the original context). Such subdivision raises the questions of which species demonstrate which forms of innovation, which abilities are necessary for each type of innovation, and whether specific forms of innovation rely on similar underlying mechanisms across species. The value of such subdivision will depend upon their bonds to underlying mechanisms and/or to functional consequences. Moreover, for categories to be useful when studying the evolution of innovativeness, it is important to use criteria that can be objectively measured in a wide range of taxa.

## 3. Approaches to the study of innovation

Innovation is often conceptualized as rare, although rates of innovation vary between species, with humans being unusually innovative [8]. Certainly innovation may be rarely observed among many animals, and this rarity can present a problem for studying innovation, since repeated occurrences allow patterns and processes to be elucidated. Broadly, animal research has addressed this issue in two ways: through observational studies of spontaneous innovations (‘innovation counts’) or through the study of innovations prompted by the presentation of novel problems or situations by the researcher (‘innovative problem-solving’). Parallel approaches are found in human research. Both animal and human research can be conducted under controlled, laboratory conditions or in field settings, which in humans often means within schools or businesses.

In animals, the spontaneous innovation count approach involves intense surveys of behaviour, either based on published literatures within taxa where there is a tradition of reporting innovations (e.g. birds and primates; this issue: [20,49,50]), or long-term direct observational surveys of behaviour in free-ranging or semi-captive populations (e.g. this issue: [34]; see also [59]). These approaches have been combined with comparative methodologies, comparing populations, species or higher taxonomic levels, as well as used to compare rates of innovations across classes of individuals (e.g. [19,51,53,60,61]). Comparative analyses of published innovation reports have examined how innovation co-evolves with brain structure, behaviour, life-history and other traits, with path analyses now being employed to explore possible causal pathways [49,50]. The direct observational approach, closely linked to investigations of animal culture [62–64], has examined population differences in innovation rates in both wild and wild versus captive groups, also addressing environmental and social influences on innovation (this issue: [34]). Note that these methods count the number of different innovations, and thus provide an index of the variety of innovation observed, rather than the reliance on or complexity of innovation. While informative, these observational methodologies are open to the possibility of reporting or other subjective biases, although measures can be taken to account for such problems [29,61,65]. Another problem is that the number of innovations documented is often relatively low, even with extensive surveys. For example, over 2500 avian innovations and over 500 primate innovations have been compiled from published literature [20,49,53,61], an impressive amount, but subdividing innovations by category or taxonomic group reduces the numbers per division considerably, compromising fine-grained analyses [49]. Furthermore, in comparative surveys of innovation in primates [61], relatively few species are recorded as innovators. This could reflect innovation being taxonomically restricted or could be a by-product of the survey method, which may be biased towards recording those innovations most salient to human observers, with those reported being in reality only the ‘peak’ of the innovations performed. In humans, there are parallels to both the database and direct observational methodologies for studying spontaneous innovations, for example comparing innovation rates using patents, or observational case studies within particular businesses [35]. While these observational studies in animals and humans allow real-world innovations to be investigated, providing external validity, the reliance on observational methods and spontaneous innovations that may only rarely be expressed or detected can compromise the power to determine underlying processes.

In contrast, presentations of novel problems by experimenters enable repeated instances of innovation to be observed in a short time span. This approach, now often termed ‘innovative problem-solving’ [31], has a long history in comparative psychology. A typical evoked-innovation study involves introducing a new problem to an individual, or, less often, to a group, allowing study of psychological processes, the adaptive consequences of innovation, and social transmission. The method also allows experimental manipulation of factors hypothesized to impact innovation, although this is still relatively uncommon in animal work at least [66]. In animal work and much research with children, the task often involves extraction of a reward from a puzzle. This method has been applied to numerous animal species in both captivity and the field, including fish, birds and mammals (review: [31]), and appears in several studies within this theme issue [20,37,67–70]. A particularly interesting subset of studies attempts to uncover the processes underlying spontaneously observed innovations with presentations of analogue tasks in the wild or captivity, such as monkey potato washing [13,71], tit milk-bottle opening [12,72–74], rats diving for molluscs [75] or finches opening sugar packets ([76,77]; issue cover image). Evoked-innovation studies bring their own problems, particularly in ensuring that the task has relevance to ‘real-world’ innovation in natural environments while still being novel, that the task captures general propensities rather than idiosyncratic performance on one particular problem, and in ensuring that the task is fair to the motivation, perceptual and motor capacities of different individuals or taxa. These are familiar problems to comparative psychologists broadly and within our fields too, but they do not always receive the attention they deserve (see e.g. [78] and commentaries thereon). Like studies of spontaneous innovation, there are measures to address such problems [79], and several additional measures are suggested in papers within this issue (see [18,37,66,80]). For example, presentation of a variety of tasks that differ in difficulty can ensure a reasonable number of solutions are observed and that the consistency of innovativeness across tasks and contexts is established. Using task presentations allows innovation to be easily observed and recorded. A major challenge is to establish the correspondence between innovations produced in the presentation of novel tasks and innovations noted during observational analyses of monitored populations. For example, in captive studies hunger and food rewards may force goal-directed innovation, with the problem and solution pre-defined by the task presented (this issue: [18]). In the wild, animals may have many behavioural alternatives available to them besides innovation. However, similar processes have been claimed to underlie innovativeness in novel problem-solving tests and in comparative analyses, leading to the conclusion that the problem-solving approach is indeed a useful paradigm for studying innovation [31].

Studies of innovative problem-solving provide an illustration of the growing cross-fertilization between animal and human literatures (e.g. [2,9,10]). Developmental psychologists have adopted paradigms used by animal behaviour researchers to examine innovation. Two clear examples appear in this theme issue [67,69]. Beck *et al*. [67] use a task originally presented to New Caledonian crows *Corvus moneduloides* (the hook task; [81]) to investigate children's innovative abilities in the same domain (the ‘floating peanut’ task has been used in a similar way: [82,83]). The hook task requires an individual to manipulate available resources (bend a piece of wire such as a pipe cleaner) in order to manufacture a tool to retrieve a bucket from a tall narrow tube. Variance in performance shows that the task is challenging, but not impossible for young children and crows, leading to questions about the differences that underpin this individual variation. Surprisingly, even children approaching 8 years of age have difficulty inventing the solution themselves, but young children readily solve the task by watching an experienced model, showing that the motor acts can easily be performed. Such results have led to the proposition that children are poor tool innovators [43], with social learning masking this deficit (for a similar conclusion in animals, see [34]). A second paradigm, ‘artificial fruits’ [69], has proved valuable across the fields of developmental psychology and animal behaviour and has allowed comparative study of chimpanzees and children (e.g. [84]). Artificial fruits are extractive foraging problems, aiming to replicate fruits with defences such as a husk that must be removed to reach a reward. The fruits can be designed with multiple solutions to reach the food, and also so that cumulative actions (such as creating new tools) can lead to better rewards. Such manipulations allow researchers to discover if similar intrinsic or contextual factors underpin the innovation seen in different species.

## 4. Social aspects of innovation

The aforementioned work on innovative problem-solving, and indeed much of the work on spontaneous innovation counts, has focused on what Sterelny (this issue: [80]) calls ‘games against nature’: solving ecological, not social problems. Sterelny [80] makes the important points that innovation also occurs in the social domain, that these social innovations may be particularly important, and even ‘ecological’ innovations occur in a social setting and have social consequences. As examples, competition may dampen the benefits of innovating if individuals cannot protect the pay-offs of their innovation, or observing others achieve rewards may promote extended exploration despite no immediate personal rewards. Thus ‘social’ and ‘ecological’ problems may not be separable [85]. Muthukrishna & Henrich (this issue: [48]) make an even stronger claim: that human sociality and social learning have driven our innovativeness and IQ, criticizing the view that innovations are the products of unusually inventive individuals or require causal understanding. Their emphasis on the importance of a diversity of experiences in recognizing and facilitating innovation (‘prepared minds’) is also found within the animal innovation literature [86]. Animal and human work does illustrate that groups and social settings are major influences on novel problem-solving [15,37,87,88]. For instance, the impact of group size and composition on novel problem-solving efficiency and innovation has been modelled and studied in human groups, with membership diversity playing an important role in generating innovations [48]. Interestingly, well-connected networks can hamper independent exploration and the solution of difficult problems [89], because lower quality solutions propagate readily, although Muthukrishna & Henrich [48] doubt human social networks are currently sufficiently well connected for this to be an issue. In animals, research on captive and wild groups of birds has reported both facilitatory and inhibitory effects of conspecific presence on novel problem-solving efficiency [90–93], and a recent model suggests that the precise effect of diversity in group composition on animal innovation will be complex [94].

Perhaps the closest contact between animal and human work related to innovation has been in studies of social learning, culture and the spread of information through groups. One observation that has promoted interest in animal innovation has been the limited spread of apparently beneficial innovations in groups [95], a phenomenon also noted in humans [6]. One idea is that innovator identity determines this spread. For example, if peripheral individuals are forced to innovate [51], much innovation may go unnoted by group members. Recent years have seen a surge of interest in the transmission of information through social networks, bringing a range of novel tools to track the identity of innovators and the cultural transmission of innovations in large populations (e.g. humans: [96–98], cetaceans: [99] and birds: [100]). Such demonstrations of the diffusion of innovations are also important because they illustrate that new behaviours can be attended to and are relevant to individuals within the population. Moreover, as Tebbich *et al.* (this issue: [18]) note, innovations are thought to have the greatest evolutionary consequences when they spread.

Appropriate innovation is also thought to be key to cumulative cultural evolution, another defining human characteristic, where a careful balance must be struck between faithful social transmission (to minimize loss of previous innovations) and innovation (to minimize stagnation and allow adaptive change; [101,102]). Lane (this issue: [88]) emphasizes how human innovation is caught in a positive feedback dynamic in which new artefacts are designed, and social organizations and novel patterns of human interaction are established to exploit and proliferate the use of these artefacts, which leads to the generation of new functionalities to the artefacts, leading to further new products. Similar auto-catalytic loops have been suggested with respect to human brain enlargement [48] and the evolution of innovativeness in animals [17]. Thus social learning and innovation are closely linked fields.

Many of the contributions in this issue reflect on the balance between social learning and innovation ([18,37,49,66–68,80], for further discussion of the relationship see also [103]). For example, Caldwell *et al.* [68] use laboratory studies of human cultural evolution to examine the rate, type and efficacy of innovation, manipulating task demands and finding a shift between the relative reliance on social versus asocial learning. Flynn *et al.* [69] examine the prevalence, effect and development of young children's preferences for using social or asocial learning. Three-quarters of children and adults chose to learn socially, when presented with an option to learn either socially or asocially. Such a preference could be one of the fundamental differences between the innovation demonstrated by humans and animals, with humans wishing to build on the behaviour of others (which may result in faithful copying, or novel modification), while animals' innovation usually occurs through individual endeavour. As humans, we may have different demands on us—related to following social norms—which impinge on our motivation to innovate, not on our capacity. However, 5-year-old children who selected asocial learning were found to be highly efficient at the task, showing that by 5 years children are selective in choosing a learning strategy that is effective for them. Such findings inform the growing interest in strategies or biases that individuals can use to identify and acquire beneficial innovations [104].

## 5. Innovativeness, cognition and intelligence

Innovation has frequently been regarded as a marker of human and animal intelligence, and to depend on domain-general cognitive abilities [7]. Indeed, the ability to solve novel problems and to innovate appears in definitions of intelligence [48,105–107], which means that, for some, innovativeness is a defining feature of intelligence. Perhaps because of these expected links to intelligence, combined with the assumed rarity of innovation, human inventors have received celebrity status, as Muthukrishna & Henrich discuss [48]. Animal innovators have also been celebrated, such as Imo, the Japanese macaque first observed sweet potato and wheat washing [13], described as a ‘monkey genius’ [108] and, more recently, Betty the New Caledonian crow [81]. Thus the term ‘innovation’ can carry an expectation of sophisticated cognitive processes. However, several authors in this issue, as well as elsewhere, note that simple cognitive processes as well as non-cognitive processes have been neglected as relevant in our understanding of innovation [18,48,66,78,109,110]. Here, we briefly review these disagreements and present our own view.

Our working hypotheses are as follows: (a) Multiple processes underlie innovation. These processes include cognitive processes (such as associative learning) and non-cognitive processes (such as perception). Different processes are likely to be involved in different instances of innovation, while some general conserved processes may underpin almost all innovations. The processes demonstrated or suggested to impact innovation are very numerous, including neophilia, neophobia, exploration, stimulus generalization, motor diversity, inhibitory control, persistence, individual learning, curiosity, insight, creativity, causal reasoning, analogical reasoning, divergent thinking, conservatism, functional fixedness and the endowment effect, as well as numerous social and environmental influences (e.g. [11,18,26,28,31,46,66,111]). Some of these processes overlap or have definitional difficulties [43], and several authors in this issue discuss their relative role [18,34,68]. (b) Simple processes [112] are likely to be common and important, even in human innovation. That is, cognitive sophistication is not a necessary condition for all innovation. However, complex processes may be essential in some instances of innovation or particularly important in some taxa. (c) Differences in innovative performance do not necessarily reflect differences in innovative ability. The social and physical environment, as well as individual phenotype, will shape the costs, benefits and constraints on innovation, and thus its performance. For this reason, rarity of innovative performance will depend on many factors and need not indicate exceptional abilities or that the innovation is beyond the abilities of an individual [113]. (d) Innovativeness is relevant to fitness and has macroevolutionary consequences [23]. Given this, it is likely that selection will shape processes that determine innovation. Selection could shape novel, derived cognitive processes, but equally may act elsewhere, such as by increasing neophilia, tolerance to unrewarded acts, motor flexibility, or by changes in motivational responses or perception. (e) While many innovations may have direct functional and evolutionary effects [18], innovativeness may be an indicator of underlying propensities, and it is these propensities that are under selection. That is, some innovative acts may be by-products that indicate underlying propensities but the acts are not themselves of functional importance. In sum, empirical evidence is required to determine the degree to which simple or complex cognitive abilities underlie different cases of animal and even human innovation, and cannot be assumed on the basis of the assumed or apparent complexity of a task.

A complete understanding of the relationship between innovation and cognitive abilities requires that certain major questions be addressed: (1) is there a trait [114] of ‘innovativeness’, for example do species and individuals consistently differ in their propensity to innovate? If so, (2) is innovativeness a marker of general cognitive ability, or are its underpinnings found in other individual-level traits, or even group-level or cultural traits? (3) Is innovativeness an adaptation, such that the traits that underpin innovation evolved specifically to promote it? Conversely, is innovativeness a by-product of selection for other characters, and, if so, is it adaptive? Griffin [66] and Sol *et al*. [50] make the case in this issue that innovativeness is an emergent phenomenon, while Sol [115] also argues that innovativeness is an exaptation. No one answer will be entirely correct, since different cases or classes of innovations may have different underlying causes and consequences, and because, even if innovativeness is an adaptation, all forms of innovation will nonetheless likely draw on other capabilities too.

A great deal of data speak to the first two questions. Within-species and across-species analyses suggest consistent differences in innovativeness. Within-species, consistent individual differences in problem-solving ability are reported in guppies and great tits, for example [116,117], and multiple studies find that individual characteristics, such as neophilia or exploratory behaviour, predict innovativeness [31,70,111]. However, several studies in the current issue indicate the difficulty of reliably identifying individual differences in innovativeness [48,67,70]. Consistent individual differences can result from many sources, including genetic predispositions, developmental environment and life-history traits [118]. In this issue, Quinn *et al.* [70] present the first heritability estimates for innovative problem-solving in a wild animal population (great tits) and find very little if any additive genetic variance. Instead individual differences were partly explained by the quality of the developmental environment and by cohort effects, suggesting an important role of plasticity in determining problem-solving performance. The generality of these effects will only become clear when many more studies repeat this approach across different tasks, other populations of great tits, and for different species. Across-species, taxa differ in innovation rates [19,53,60,61,111]. An excellent example is provided by Lefebvre *et al.* (this issue: [20]), who show using comparative phylogenetic methods that a particular group of birds, the Darwin's finches, renowned for their innovation, are part of a larger clade of unusually flexible birds that all exhibit high rates of innovation compared with other neotropical clades. These same birds are characterized by high rates of speciation, and Lefebvre *et al.* argue that variation in innovativeness may explain variation in speciosity, such that adaptive radiations are favoured when the ancestral stem species were flexible. Again, multiple factors may influence taxonomic differences in innovativeness, and teasing apart these contributions and the relations between within- and across-species correlations are important open questions [109,111].

Regarding the second question, a growing set of correlational data link innovativeness and various cognitive abilities. In humans, the link between innovation and intelligence is regarded as long established [5,119], although Muthukrishna & Henrich (this issue: [48]) make an important case to reconsider the direction of causality. A particularly relevant paper in the current issue is that of Beck *et al.* [67] who demonstrate that, while divergent thinking, inhibition, working memory, attentional flexibility and ill-structured problem-solving do not predict tool invention in the hook task in mid-childhood, it was predicted by a proxy for general intelligence (receptive vocabulary scores). It remains unclear whether the link highlighted by Beck and colleagues transfers across other innovative contexts, or across species. In animals, within-species analyses have documented correlations between innovative problem-solving and measures such as learning speed [54,66,120,121]. Across-species analyses have documented correlations between innovation counts and laboratory tests of learning in birds and primates [122], with composite measures of cognitive performance in the laboratory [61], and with experimental inhibitory-control tasks [123]. Within primates, innovation counts covary with counts of social learning, tool use, extractive foraging and tactical deception, while avian tool use and innovation counts covary [49,61,122]. These results suggest that a suite of traits have evolved together, and, to the extent that these counts can be regarded as indicators of general cognitive abilities, are consistent with a general intelligence account. Social learning, tool use, extractive foraging and tactical deception have all been proposed as indicators of general cognitive abilities, although again this is debated [124]. Our view is that a particularly interesting aspect of innovation counts is that they provide an indicator of the *variety* of behaviour, and thus can be considered an estimate of behavioural flexibility. Innovative taxa have been found to be more likely to become established when translocated to new environments with novel challenges [38], again possibly owing to their greater behavioural flexibility. Thus although the evidence is correlational, there is wide support for the idea that cognitive performance may underlie differences in innovation counts.

Animal innovation counts have also been found to covary with measures of brain enlargement, again with parallel results in birds and primates, increasing confidence that these relationships are robust [49,122]. This could potentially be taken as further evidence for a link between innovation and cognitive traits, but this rests upon the assumption that large brains facilitate enhancements in cognitive performance. Instead, we see these data as supporting the view that brain enlargement has functional consequences for behaviour. Although this view has been questioned [125], the large number of correlations between brain volume and behavioural measures require explanation, especially given the significant costs of brain enlargement [61]. Moreover, we note that linking innovation and cognitive performance does not rely on demonstrating correlations with brain size: instead, most relevant are the aforementioned relationships between innovation, learning and other cognitive measures. In humans, a substantial literature has examined links between neural measures and measures allied to innovation [106,107]. Striedter [126] notes that changes in brain anatomy over recent human evolution support a relationship between innovation and cognitive performance, although in this issue Sterelny [80] presents an opposing view, and Muthukrishna & Henrich [48] argue that the exceptionally large human brain is partly a result of feedback processes between sociality and culturally transmitted cumulative innovations.

We concur with others that simple explanations and mechanisms are often overlooked in studies of animal and human innovation [18,66], just as they often are in studies of cognition in general [112]. Moreover, simple explanations are exciting since they extend the possible breadth to which work on innovation applies. For example, we are pleased that concepts from the animal innovation literature have been used to inform work on host selection in insects [47]. However, complex mechanisms need not be neglected, particularly in human technological innovation, and empirical investigation is required to determine the mechanisms underlying innovation, and indeed what constitutes a simple or complex mechanism. Various means can be used to establish underlying mechanisms, and several contributions to this theme issue argue for new or improved methodological approaches [18,66,68,70]. Experimental task manipulations can determine the cognitive processes operating and eliminate others, as well as address other possible determinants of innovation, such as individual state, experience, competitive regime, environmental variability or other environmental variables. For example, Taylor *et al.* [127] found evidence that perceptual-feedback-based operant learning underpinned birds' solving of a string-pulling task, a task once thought to involve ‘insight’. The techniques of behavioural neuroscience can also be applied, measuring or manipulating proposed substrates of innovation. Another possibility is to manipulate innovative propensities [128–130] or the cognitive processes thought to underlie innovation [18] using training, psychopharmacological, or other procedures. The subdivision of innovation into constituent processes may help here [18]. For example, groups of individuals could be trained in differing conditions, such as training their inhibitory control, or a stronger confidence to reject the social norm, to establish the resulting effect on their innovation. There is already evidence that animals can ‘learn to innovate’ [130], and extensions of this approach to controlled, large-sample studies would be welcome. Such studies would not only allow investigation of psychological processes, but by experimentally impairing innovation or its underlying processes the functional impact of these manipulations could also be measured.

Finally, we note that the sophistication of the cognitive processes involved need not predict the adaptive value of the related behaviour, and both ‘simple’ and ‘complex’ processes may underlie ecologically and evolutionarily significant innovations, although certain processes may have particularly significant eco-evolutionary impact (this issue: [18]). Sterelny [80] makes a similar observation when comparing the experimental work on human innovation in this issue with the theoretical work in archaeology. Clearly, many avenues are open to address these questions.

## 6. Promoters of innovation and innovativeness

An issue of great interest is how best to promote innovation and innovativeness. Resource availability has been a major area of interest in both human and animal research, with a focus on the contrasting roles of necessity, opportunity and free resources. For example, in business both an excess (‘slack’) or limited resources can promote innovation, and may have different effects on different kinds of innovation [131]. Within the creative arts too, the role of financial need has been contrasted with that of spare resources or advantage, and both have been noted to drive or limit creativity and innovation [132]. In animals, Kummer & Goodall [15] suggested that ‘free time’ (spare energy or time) may promote innovation, while also pointing to innovation driven by need, such as low social rank or changing environments.

While ‘necessity is the mother of innovation’ has been supported in several animal studies [11,31], overall evidence is mixed for the ‘necessity hypothesis’ (i.e. that lack of access to or scarcity of resources prompts innovation; review: [31]). Koops *et al.* [133] examined data from capuchins, orang-utans and chimpanzees and found no support for the idea that necessity (here, shortage of preferred foods) drove tool use rates, but did find support for an alternative ‘opportunity’ hypothesis, i.e. that encounter rates with resources increase tool use. Koops *et al.* use this data to argue that opportunity availability will also shape tool invention, given the appropriate social setting and cognitive capacities. Similarly, the necessity hypothesis was not supported in captive orang-utans in comparison to wild orang-utans [34,134]. van Schaik *et al.* (this issue: [34]) argue that the orang-utan data support a variant of Kummer & Goodall's [15] ‘free-time’ hypothesis, namely that the lack of predation in captivity allows long undisturbed periods of independent exploration. Modelling the net pay-offs of innovative behaviour in various contexts according to the time, effort or risk invested may help to define more precisely the predictions of these various hypotheses [28]. Relatively subtle influences may also shape innovation [68]. For children, a more informal environment appears to facilitate exploration and as a result tool invention. Sheridan *et al.* (this issue: [37]) show that informal learning environments, such as museums, facilitate tool exploration and invention. Further, conveying ownership over materials may encourage successful tool selection at earlier ages than has previously been demonstrated. We thus foresee no reason to expect innovation to be caused by a single factor. The proverb ‘necessity is the mother of invention’ was first coined for humans, but clearly not all human innovation is driven by necessity [88]. Rather, much innovation arises when people see new ways of exploiting existing technology and thereby instantiate new functionalities. The same is likely to be the case for other species.

Griffin (this issue: [66]) discusses the usefulness of categorizing innovations and making specific predictions on relevant individual-level predictors of innovativeness, for instance based on the novelty of the context. Indeed, neophilia should be a stronger predictor of innovations involving a novel situation compared with those stemming from motor adjustments in a known context, or to use van Schaik *et al*'s categories [34], neophilia should predict the frequency of ‘novelty-induced’ innovations but not ‘failure-induced’ or ‘accidental’ ones. The observed inconsistencies regarding the relations between individual characteristics and innovativeness may imply that some individual-level characteristics exert a probabilistic influence on the likelihood of innovation, rather than being necessary for innovation. These findings are equally consistent with the ‘emergent property’ view of innovation [50,66,70], whereby innovativeness is not an evolved trait itself but rather emerges from a set of underlying traits (e.g. boldness, neophilia, high propensity to learn, etc.). Possessing only one trait associated with innovation might not always be enough to facilitate innovation [18], potentially leading to inconsistent relationships between the variables depending on the presence or absence of other traits. Under this scenario, phylogenetic lineages where the expression of innovations has consistently led to fitness benefits could have experienced selection for several traits simultaneously.

## 7. Concluding remarks

The differences of opinion that we have outlined above may have arisen owing to the conceptual approach taken by different disciplines. For example, psychologists have tended to focus on evoked innovations produced through the presentation of novel tasks, with investigation of potential facilitators or inhibiting factors, as their primary focus has been on the cognitive mechanisms and contextual factors underpinning innovation. In comparison, anthropologists have examined the cultural factors related to innovation at the group level, including the social network of differing communities and how this has influenced technological change. Behavioural ecologists tend to focus on the evolutionary causes and consequences of innovation, and thus on innovation as a functional product rather than its underlying processes. However, this theme issue, as well as work elsewhere (e.g. [10]), shows that the questions asked regarding innovation have begun to cross disciplinary boundaries, and the utility of these approaches. For example, knowledge about the cognitive processes underlying innovation, their costs and their interdependence with other traits, allows a behavioural ecologist to consider what must evolve for innovation to happen. This crossing of field boundaries is demonstrated by the fact that a number of the papers in this issue are co-authored by multi-disciplinary teams (e.g. [67,69]).

We believe that such collaborations will benefit the field both in terms of direct findings from their endeavours, but also in terms of fertilizing ideas across disciplines. This may result in consensus regarding definitions, terminology, experimental techniques and analytical protocols, and the commentators on the current theme issue point to some important gaps [66,80]. However, equally, different protocols and foci are appropriate for different questions. These synergies further our understanding of innovation, such as the role of curiosity or necessity. This cross-disciplinary dialogue also allows comparisons of where theories may diverge along taxonomic lines. For example, humans build on the innovations of others to produce sophisticated behaviours and technologies through incremental modification, sometimes within long-term planned initiatives, while such cumulative innovations are non-existent in the animal kingdom. All the papers in this issue highlight areas of future exploration. Compilation of anecdotal reports, extensive observation of groups, experiments in natural locations and experiments in laboratories will all have a place in our understanding. We look forward to new research and new findings in the coming years.
